# CheekAge: a next-generation buccal epigenetic aging clock associated with lifestyle and health

**DOI:** 10.1007/s11357-024-01094-3

**Published:** 2024-03-05

**Authors:** Maxim N. Shokhirev, Nicole S. Torosin, Daniel J. Kramer, Adiv A. Johnson, Trinna L. Cuellar

**Affiliations:** Tally Health, New York, NY USA

**Keywords:** Epigenetic age, Aging clock, Ensemble learning, Machine learning, Simulated annealing, Buccal

## Abstract

Epigenetic aging clocks are computational models that predict age using DNA methylation information. Initially, first-generation clocks were developed to make predictions using CpGs that change with age. Over time, next-generation clocks were created using CpGs that relate to both age and health. Since existing next-generation clocks were constructed in blood, we sought to develop a next-generation clock optimized for prediction in cheek swabs, which are non-invasive and easy to collect. To do this, we collected MethylationEPIC data as well as lifestyle and health information from 8045 diverse adults. Using a novel simulated annealing approach that allowed us to incorporate lifestyle and health factors into training as well as a combination of CpG filtering, CpG clustering, and clock ensembling, we constructed CheekAge, an epigenetic aging clock that has a strong correlation with age, displays high test–retest reproducibility across replicates, and significantly associates with a plethora of lifestyle and health factors, such as BMI, smoking status, and alcohol intake. We validated CheekAge in an internal dataset and multiple publicly available datasets, including samples from patients with progeria or meningioma. In addition to exploring the underlying biology of the data and clock, we provide a free online tool that allows users to mine our methylomic data and predict epigenetic age.

**Supplementary Information:**

The online version contains supplementary material available at 10.1007/s11357-024-01094-3.

## Introduction

Mammalian aging is a complex, multifactorial process characterized by molecular, cellular, and organ system dysfunction. Although aging remains a poorly understood process, the field has converged on a set of 12 hallmarks that become aberrant over time and can be targeted to shorten or lengthen lifespan in model organisms [1]. However, which of these hallmarks is the most foundational has yet to be determined. Two strong contenders are genomic instability [2] and epigenetic alterations [3]. Arguing for a combination of both, a recent study created transgenic mice that repeatedly experience double-stranded DNA breaks but do not accrue mutations [4]. These non-mutagenic breaks were reported to erode the epigenome, induce an accelerated aging phenotype, and elevate epigenetic age [4]. This latter finding is interesting in the context of some epigenetic clocks correlating with age-related health outcomes in longitudinal data and responding to interventions in clinical trials [5].

Myriad epigenetic aging clocks have been developed, and they vary in their tissue-specificity, correlation with chronological age, ability to capture health, and test–retest reliability across replicates. In terms of algorithms that incorporate information from other biomarkers, the published next-generation models GrimAge2 [6], bAge [7], and DunedinPACE [8] represent the state of the art. While innovative, these models were built using methylomic data derived from blood, which can be invasive, unpleasant, and challenging to collect in a home setting. Currently, the biohorology field [9] is lacking a published clock optimized for cheek swabs, a sample type that can be painlessly and easily collected in a variety of environments. Previous research suggests that buccal tissue is a viable sample type for epigenetic age prediction [10, 11].

To address this gap, we used an innovative computational approach in conjunction with a MethylationEPIC dataset paired with health and lifestyle questionnaire data from more than 8000 diverse adults. As presented below, the result is a unique buccal clock optimized for estimating an epigenetic age value that is associated with a plethora of lifestyle and health factors.

## Methods and materials

### Cohort selection and survey

We selected 10,000 volunteers from a larger cohort of over 25,000 who filled out an online questionnaire and consented to collect and send in a buccal sample. We selected volunteers with valid United States addresses while maximizing demographic diversity (chronological age, gender, and race/ethnicity) as well as various lifestyle and health (see Table S1). Of the 10,000 kits sent, 8045 samples (including 190 replicate pairs) were returned and passed all quality control checks. For each of the 8045 samples, we collected responses to 11 lifestyle- and health-related questions focused on self-rated health, self-perceived aging, sleep quality, stress levels, social satisfaction, fraction of a diet that is plant-based, exercise frequency and intensity, smoking history, weekly alcohol consumption, relative immune health, and BMI based on self-reported weight and height. Beyond gender, we asked three demographic questions: date of birth, race/ethnicity, and education level achieved. We also predicted sample sex using methylation intensity across chromosomes and estimated cell-type proportions via the methylation data directly (described in the Supplementary Methods). To calculate correlations to lifestyle, health, and demographics, survey responses were scaled to a value between 0 and 1. Binary demographic variables were arbitrarily assigned either − 1 or 1.

### Sample collection

A total of 10,000 volunteers were mailed a buccal collection kit, which consisted of two VARE (Shenzhen City, Guangdong, China) flocked swabs (cat. no. VF106-80), two Mawi DNA Technologies (Pleasanton, California, USA) iSWAB-Discovery Human DNA collection devices (cat. no. ISF-T-DSC), customized instructions, and mailing pouches. Volunteers were asked to perform two collections within a 24-h period, send back both replicate samples, and register their kits. Collection instructions are provided in Supplementary Table 1.

### EPIC array

Samples were preprocessed at Tempus Labs (Peachtree Corners, Georgia, USA) according to Illumina’s (San Diego, California, USA) protocols for MethylationEPIC array preprocessing and loaded onto MethylationEPIC arrays. While most samples were run using the combined DNA from both collection devices to improve yields, we manually selected samples from 190 diverse individuals in our cohort to be run as replicates.

### Constructing the CheekAge clock

Briefly, the EPIC arrays were preprocessed using the minfi (v 1.44.0) [12] preprocessing pipeline. CpGs were then filtered until only approximately 200,000 higher-quality CpGs remained. These higher-quality CpGs were then clustered based on their methylation pattern across the entire dataset, and the top 10,000 clusters were calculated by averaging the CpGs in each cluster, resulting in the set of independent variables for model training. A simulated annealing [13] approach was used to train linear models optimizing for model accuracy, significance of the correlation between delta age and lifestyle/health factors, and model complexity. Finally, the model training was repeated 1098 times, and a weighted mean of the top 100 scoring models was used to predict CheekAge. Please see the Supplemental Methods for extensive details on preprocessing, CheekAge clock construction, clock metrics used for evaluating the clock, and the key functions and arguments used.

### Evaluating CheekAge in external, publicly available datasets

Raw MethylationEPIC data was downloaded and CheekAge predictions required averaging of CpG M values for 10,000 CpG clusters. Inputs were then used to predict CheekAge using the weighted 100 ensemble model calculations*.* Briefly, we reprocessed 10 publicly available EPIC array datasets including an external buccal dataset (GSE111165), a blood methylation dataset looking at SARS CoV-2 infection (GSE167202), a human skin dataset from progeria patients (GSE151617), a dataset exploring accelerated aging in childhood cancer survivors (GSE197674), a meningioma dataset (GSE183647), a primary human fibroblasts dataset (GSE179847), a rectal sample dataset (GSE216024), a colorectal cancer dataset (GSE199057), a dataset looking at cultured epithelial cells antagonized with rhinovirus (GSE172365), and a melanocytic nevi dataset (GSE188593). Significance of association with delta age was calculated using linear models considering available confounding variables. We describe the specific datasets and linear model tests in the Supplemental Methods section and Supplementary Table 10.

## Results

### Cohort information and chronological age trends

We started by collecting buccal DNA samples and digital questionnaire responses from 8045 volunteers. Two replicate DNA samples per subject were obtained, allowing us to measure variability in methylation caused by biological and technical noise for a subset of 190 participants with sufficient DNA collected. The 8045 EPIC samples were used in combination with the lifestyle and health information to build CheekAge, a next-generation epigenetic aging clock that correlates with chronological age, lifestyle, and health. Importantly, our cohort of 8045 samples is the largest adult buccal methylomic cohort that we are aware of, includes a chronological age range of 18 to 93 years (Fig. S1a), a similar distribution of sexes (Fig. S1b), and is diverse (Fig. S1c). Please see Supplementary Table 1  for detailed demographic information and Supplementary Table 2 for all questionnaire responses.

We started by dissecting the questionnaire responses (Table S2), finding unique patterns across chronological age (Fig. S1d, e). All variables except sex were significantly associated with chronological age (Table S3). Compared to younger respondents, older respondents tended to self-rate their health as better (*P* < 2e − 16), to feel younger than their chronological age (*P* = 2.68e − 03), to have lower stress levels (*P* < 2e − 16), to be more socially satisfied (*P* = 3.26e − 07), and to get sick less often (*P* < 2e − 16). For the categories of self-rated health, self-perceived aging, sleep quality, education, and social satisfaction, we noticed that the median chronological age for the response corresponding to moderately lower values (0.25 or 0.2) was consistently the youngest than for all other responses. Older respondents typically had a more plant-based diet (*P* < 2e − 16) and smoking was reported to be less common among younger respondents (*P* < 2e − 16). Lastly, we saw that chronologically older respondents tended to be white while more of our chronologically younger respondents identified as non-white (*P* < 2e − 16). Please see Supplementary Table 3 and 4 for additional details.

We then analyzed sex-specific differences in survey responses (Fig. S2 and Tables S3 and S4). The chronological age range for male and female respondents was the same, but the median chronological age for females was 4 years older. For males, all survey responses associated significantly with chronological age except self-perceived aging, alcohol, and education. For females, all survey responses except exercise intensity significantly associated with chronological age. Two responses showed the greatest sex-specific trends. The first was education, where education level correlated significantly with chronological age for female respondents (*P*s < 2e − 16) but not for male respondents (*P* = 0.0742). The second was BMI, where male BMI significantly decreased with chronological age (*P* = 1.83e − 08) while female BMI increased with chronological age (*P* = 9.19e − 04). Interestingly, self-perceived aging and alcohol use in males were not significantly correlated with chronological age. However, self-perceived aging and alcohol use were significantly correlated for females (*P* = 1.02e − 04 and *P* = 1.86e − 04, respectively).

### Building a next-generation buccal clock relevant to lifestyle and health

Similarly to a prior study [10], we first trained a standard penalized linear regression model using a tenfold cross validation approach. This first-generation clock correlated highly with chronological age (Fig. S3a), but delta age (epigenetic age–chronological age) failed to significantly correlate with survey factors (Fig. S3b, c). The one exception was alcohol use, which was significantly associated with a false discovery rate (FDR) < 0.002.

**Fig. 1:**
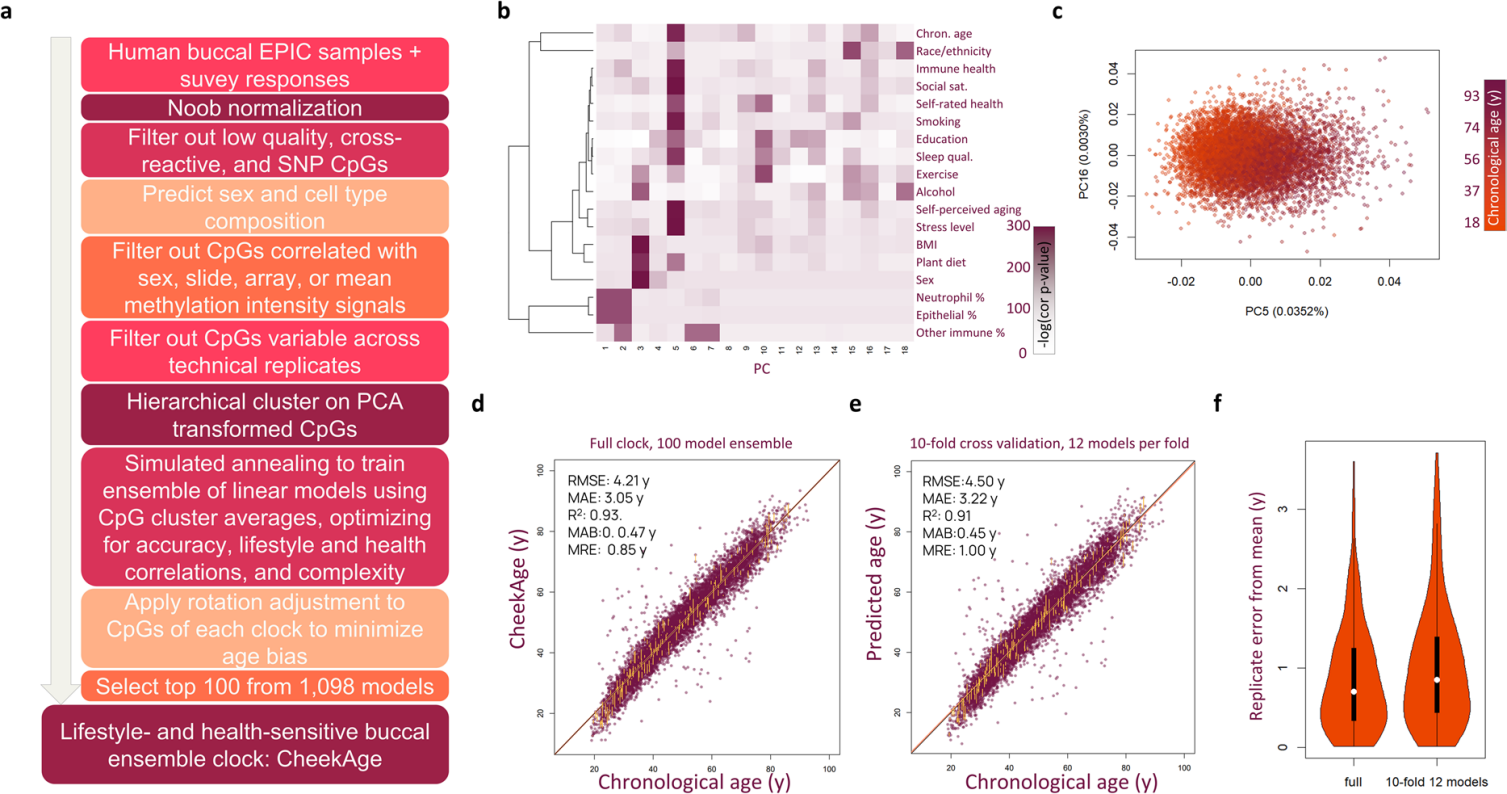
Construction of the CheekAge clock. **a** Workflow for building the CheekAge clock. **b** Clustered heatmap showing correlation of lifestyle/health, demographic, and technical variables with the first 18 principal components (PCs). **c** Scatterplot showing that chronological age signals are captured in PC5 and PC16. **d** Scatterplot showing CheekAge vs chronological age trained on the entire dataset. **e** Scatterplot showing predicted age using a tenfold cross validation approach, with 12-model ensembles trained for each fold. For **d** and **e**, RMSE (root mean squared error), MAE (mean absolute error), *R*^2^ (squared Pearson correlation), MAB (mean absolute bias), and MRE (mean replicate error from the mean) are shown. Yellow lines connect replicates taken within a 24-h period. **f** Violin plots showing the MRE for the full clock and the tenfold cross validated 12-model ensemble

To improve upon this, we created a custom objective function that included the root mean square error (RMSE) of the predicted age as well as the log of the significance of correlations between delta age and answers to survey questions. We then used a simulated annealing [13] optimization strategy along with a number of model building strategies to construct our next-generation epigenetic clock, including extensive CpG filtering to remove noisy or biased CpGs, training on clusters of CpGs to minimize noise, and using a weighted ensemble of models to further improve clock accuracy and reproducibility (Fig. 1a). As an intermediate test, we calculated the principal components (PCs) of the clustered CpG inputs and saw significant correlations between survey factors as well as technical covariates with the first 18 PCs (Fig. 1b and Table S5). All lifestyle and health factors showed strong correlations with one of the 18 PCs (Fig. 1b), and chronological age correlated strongly with PCs 5 and 16, (Fig. 1b, c). As a consequence of using a stochastic simulated annealing optimization strategy (Fig. S4a), each optimization yielded a different clock with a unique final model score. We repeated this process 1098 times for the full dataset to yield 1098 models, resulting in a distribution of final model scores, accuracies, complexities, and lifestyle correlations (Fig. S4b and Table S6). Typically, there was a tradeoff between model accuracy, complexity, and lifestyle/health correlations, with the highest-scoring and lowest-scoring models exemplifying this difference (Fig. S4c). By combining different numbers of models into an ensemble, we were able to improve accuracy, reproducibility, and correlation with survey responses at the cost of model complexity (Fig. S5). We determined that 100 models produced optimal scores, after which the improvements were marginal. Therefore, the top 100 scoring models were combined using a weighted averaging approach (Fig. S4d), to produce our final CheekAge clock. Most CpG clusters used as inputs were only utilized by a fraction of the 100 models (Fig. S4e), and each cluster included anywhere from 1 to over 150 CpGs (Fig. S4f).

To explore how the models differ from each other, we generated clustered heatmaps for all 100 models (Fig.S6). Models tended to use diverse patterns of cluster weights to predict age, and some clusters were consistently incorporated with negative or positive weights across models (Fig. S6a). Turning to the delta age predicted by CheekAge, we noticed that even though model weights tended to be diverse between models, the ages predicted were similar to the ensemble average for any specific sample (Fig.S6b). Importantly, models with the lowest optimization score and hence highest contribution toward CheekAge were dispersed among the model clusters.

Taken together, our CheekAge clock was highly predictive of chronological age, had low age bias, and a low test–retest error when training and predicting in our full dataset (Fig. 1d) or when using a tenfold cross validation approach that uses an ensemble of 12 models per fold to estimate expected error in new similar data (Fig. 1e). In the cross-validation data, the *R*^2^ was 0.91, the RMSE was 4.5 years, the mean absolute error (MAE) was 3.22 years, and the mean absolute bias (MAB), an indicator of chronological age bias, was 0.45 years. We observed a mean replicate error from the mean (MRE) of 0.85 years and 1 year in the full and cross-validated versions of our clock, respectively (Fig. 1f).

**Fig. 2:**
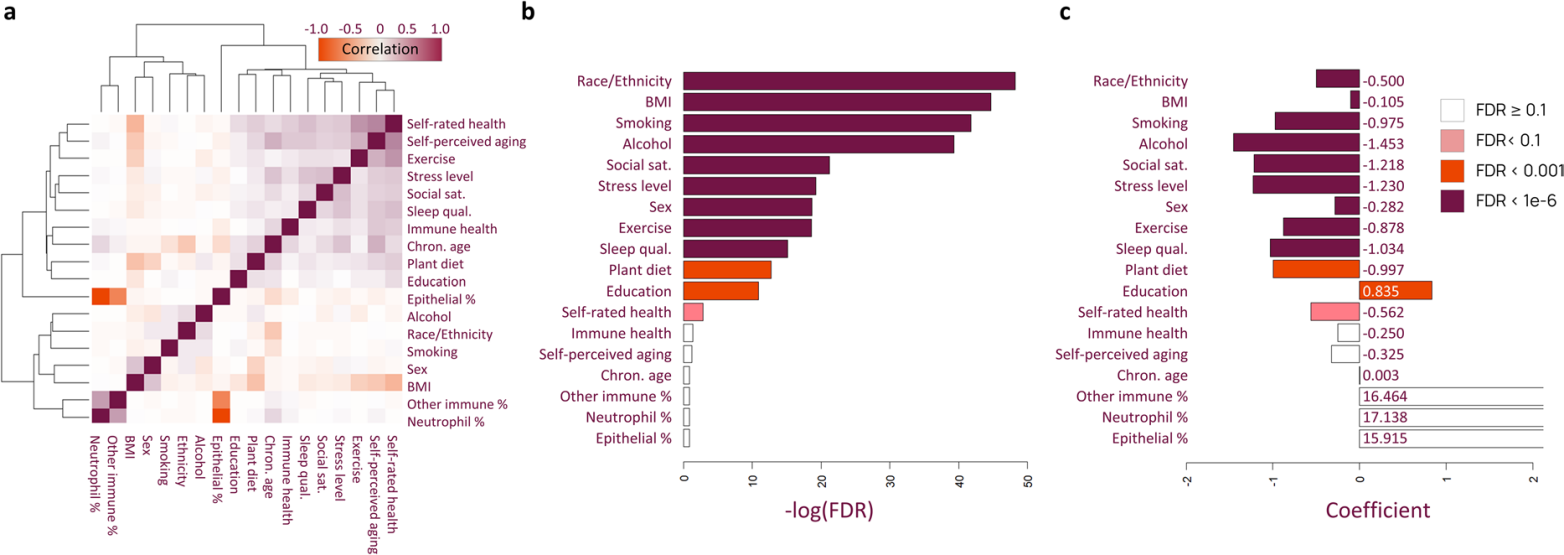
Lifestyle and health factors correlate significantly with delta CheekAge. **a** Clustered heatmap showing Pearson correlation of lifestyle/health, demographic, and technical variables across all samples. **b** Significance of each variable when modeling delta CheekAge as a linear combination of survey factors. **c** Linear model coefficients for delta CheekAge modeled as linear combination of variables

Since lifestyle/health responses were correlated with other lifestyle/health responses, demographics, and cell types (Fig. 2a and Table S7), we modeled delta age as a linear combination of all lifestyle/health, demographic, and technical variables to estimate the significance of each lifestyle factor correlation with all other factors held constant. Using a FDR cut-off of 0.05, we found that BMI, smoking, alcohol, social satisfaction, stress levels, exercise, sleep quality, and percent of diet that is plant-based were correlated with delta age (Fig. 2b). Self-rated health displayed a FDR of 0.0586. Importantly, coefficients of the linear fit for all of these factors were changing as expected, with healthier lifestyles predicted to decrease delta age (Fig. 2c, Fig. S7, and Table S8). Lifestyle and health correlations changed depending on which model in the ensemble was used to predict age, and this was largely unaffected by the relative weight of each model (Fig.S6c). Sex-specific correlations between delta age and lifestyle/health factors are summarized in Supplementary Fig. 8 and Supplementary Table 7.

### Validating the CheekAge clock

**Fig. 3:**
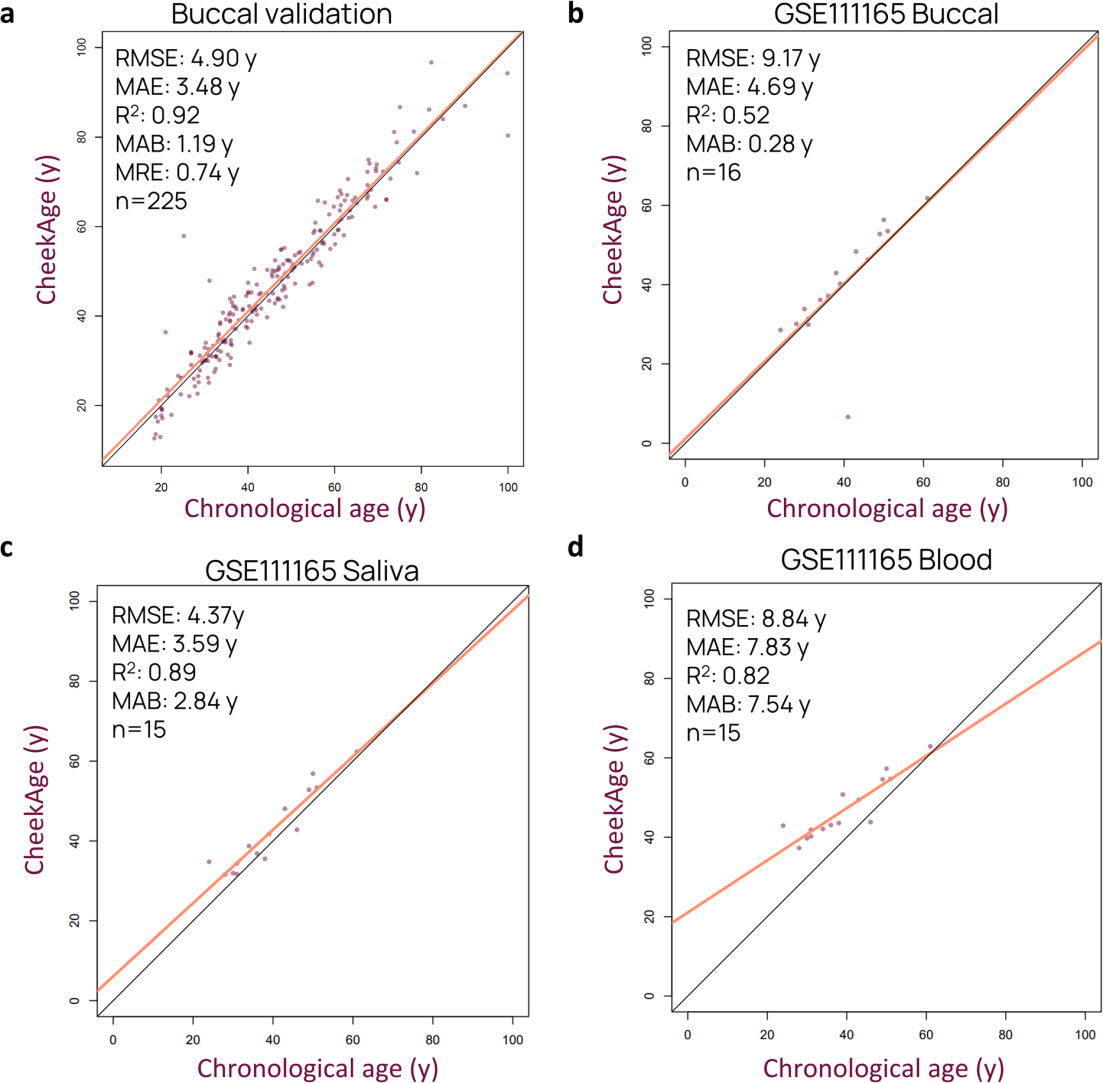
Ability of CheekAge to predict age in other datasets. **a** Scatterplot and clock statistics for a buccal validation dataset that was collected the same way as the training dataset but held out during training. The chronological age range of subjects in the validation dataset is 18–100 years. **b**–**d** Scatterplot and clock statistics for a publicly available dataset with methylation data from tissues of medically intractable epilepsy patients. **b** Buccal, **c** saliva, and **d** blood tissue predictions are shown. The chronological age range of subjects in the publicly available dataset is 24–61 years

We next wanted to test how our CheekAge clock performs in other datasets (Table S9). We started by testing its performance in an independently collected buccal dataset (*n* = 225) with an age range of 18–100 years. Our clock performed well in this dataset, with a *R*^2^ of 0.92, a MAE of 3.48 years, and a MAB of 1.19 years (Fig. 3a). We then downloaded and reprocessed a publicly available dataset [14] containing multi-tissue data from patients with intractable epilepsy. The *R*^2^ was 0.52 and the MAE was 4.69 years in buccal tissue (*n* = 16), mostly owing to one outlier that was predicted to be much younger (Fig. 3b). The accuracy metrics were higher in saliva (*n* = 15), with a *R*^2^ of 0.89 and a MAE of 3.59 years (Fig. 3c). In blood (*n* = 15), the *R*^2^ was 0.82 and the MAE was 7.83 years (Fig. 3d).

**Fig. 4:**
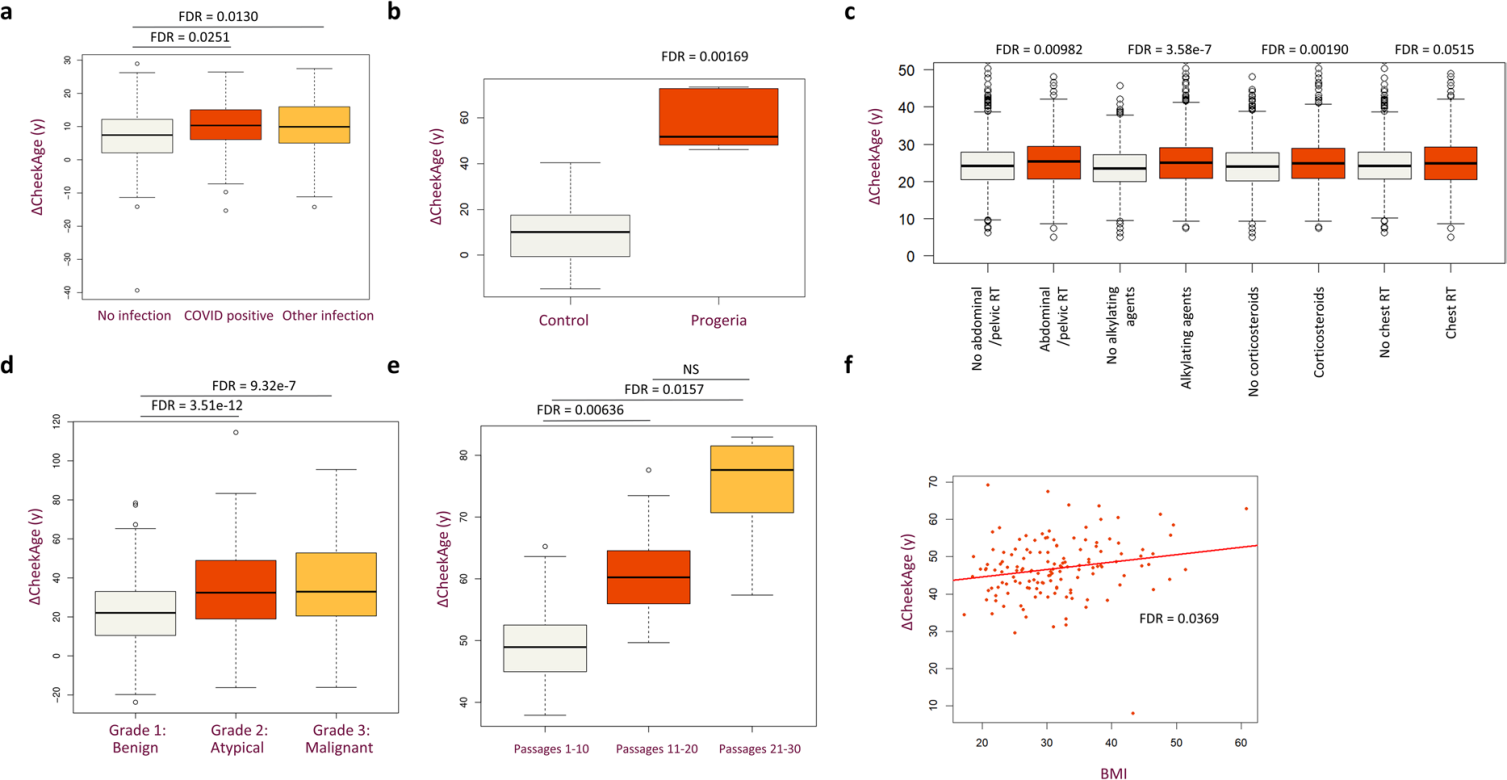
Ability of CheekAge to associate with health signals in external datasets. **a** Compared to COVID-negative individuals (*n* = 296), delta age was significantly elevated in blood from COVID-positive individuals (*n* = 164) or individuals with a non-COVID acute respiratory infection (*n* = 65). **b** Relative to controls (*n* = 27), delta age was similarly significantly increased in progeria samples (*n* = 9) in a skin dataset. **c** In a blood dataset from adult survivors of childhood cancers (*n* = 2138), delta age significantly correlated with abdominal/pelvic radiation therapy, alkylating agent treatment, and corticosteroid treatment. **d** Compared to benign meningiomas (*n* = 388), meningiomas classified as atypical (*n* = 142) or malignant (*n* = 35) are predicted to be significantly older. **e** Delta age significantly increased with passage number in fibroblasts derived from healthy people. Specifically, passages 11–20 (*n* = 49) and 21–30 (*n* = 18) were significantly higher compared to passages 1–10 (*n* = 51). **f** BMI and delta age significantly correlated with one another in colorectal samples (*n* = 140)

We also tested whether our clock was able to pick up health- and age-related signals in other datasets [15–20]. To do this, we downloaded and reprocessed six different publicly available datasets containing health and/or disease information (Fig. 4, Fig. S9, and Table S10). As shown in Fig. 4 and Supplementary Fig. 9, we observed significant correlations between delta age and SARS-CoV-2 infection (Fig. 4a), a non-SARS-CoV-2 infection (Fig. 4a), progeria (Fig. 4b), cancer survivors who underwent abdominal/pelvic radiation treatment, alkylating agent treatment, or corticosteroid treatment (Fig. 4c), meningioma (Fig. 4d), fibroblast passaging (Fig. 4e), and BMI (Fig. 4f). We also analyzed additional datasets [21, 22] which showed harder-to-interpret associations, namely a positive correlation with tumor formation but a negative correlation with colorectal cancer, some associations in rhinovirus-antagonized epithelial cells and steroid treatments, and an association with both navel dysplasia and control skin samples in a melanocytic nevi dataset (Table S10). Please see the Supplementary Methods for additional information, including which variables were controlled for in each analysis.

**Fig. 5:**
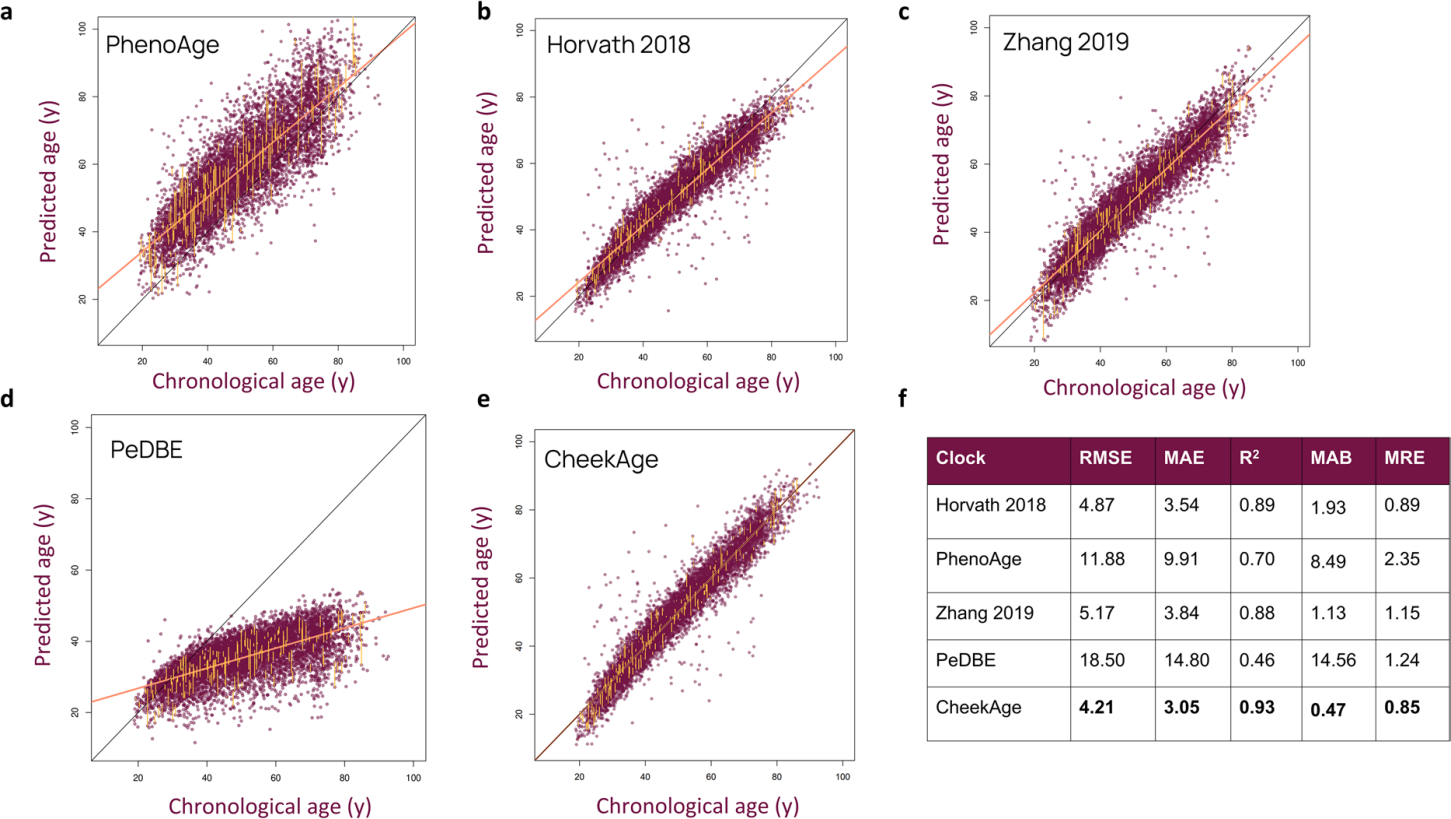
Predicted age of 8045 adult buccal samples using external clocks. Panels show chronological age compared to predicted age for **a** PhenoAge, **b** Horvath et al. [24], **c** Zhang et al. [25], and **d** PedBE alongside **e** CheekAge. Replicate samples are indicated by vertical yellow lines and the linear model fit is indicated by orange diagonal lines. **f** Summary of metrics for each model fit

We next compared CheekAge to four other epigenetic clocks for which all CpGs were available in our dataset. Specifically, we analyzed RMSE, MAE, *R*^2^, age bias, and test–retest error of PhenoAge [23] (Fig. 5a), Horvath et al. [24] (Fig. 5b), Zhang et al. [25] (Fig. 5c), and PedBE [11] (Fig. 5d) alongside CheekAge (Fig. 5e). As would be expected since none of these clocks were optimized for adult buccal tissue, CheekAge displayed the best overall performance (Fig. 5f), even after controlling for systematic age bias using the same rotation transformation applied to CheekAge (Fig. S10). All of the non-CheekAge clocks also showed dramatically poorer correlation between delta age with questionnaire responses, regardless of whether or not age bias rotation was applied (Fig. S11 ). Predicted ages and correlations of delta ages with lifestyle/health in both rotated and unrotated versions of the clocks can be found in Supplementary Table 11 and 12, respectively. Clock performance in various validation datasets, including an additional dataset [26], is summarized in Supplementary Fig. 12 and Supplementary Table 13.

### Deriving biological insights from our dataset and clock

**Fig. 6:**
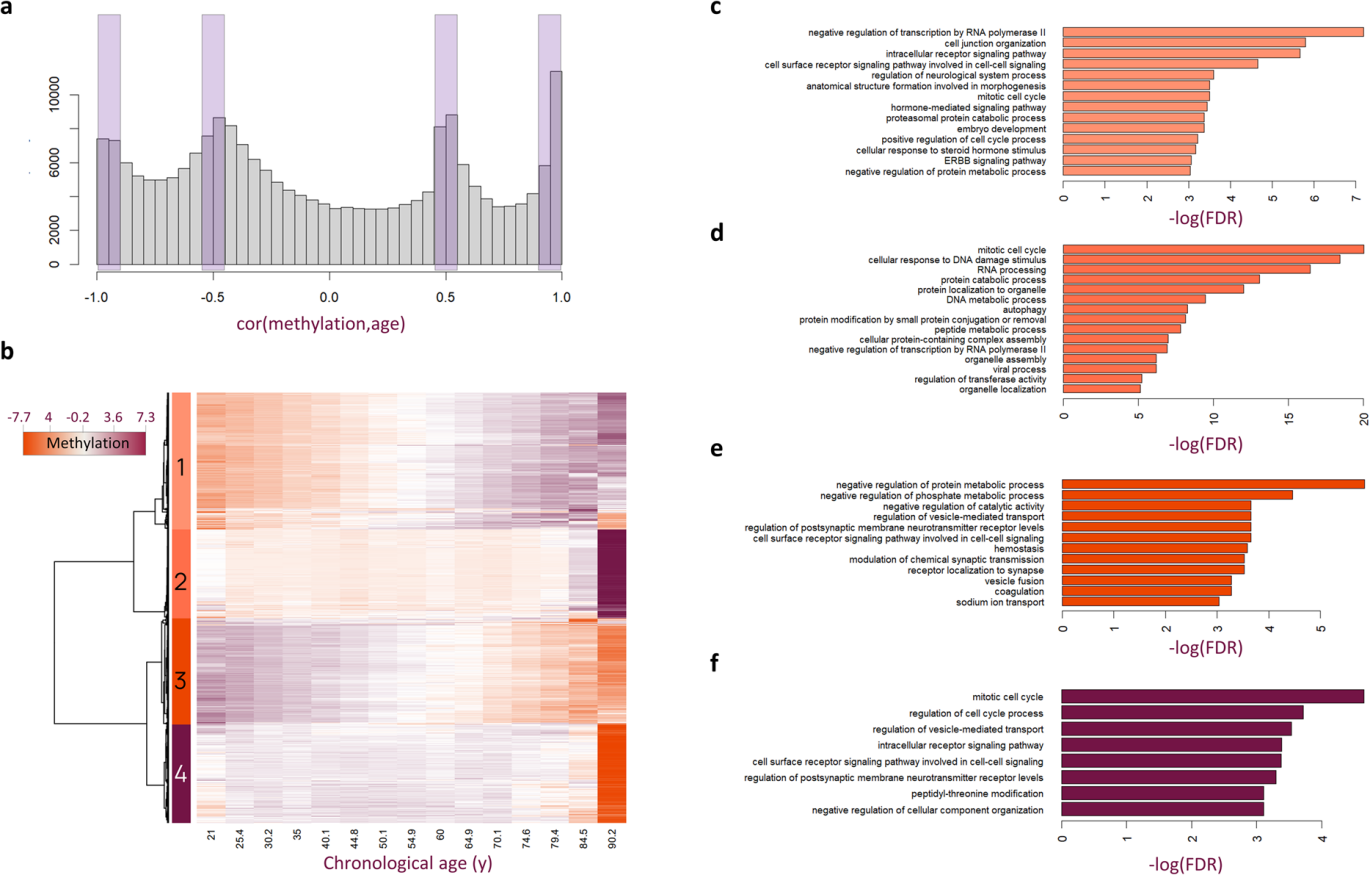
Methylation patterns are correlated with age and show enrichment for biological processes. **a** Samples were binned by chronological age and the distribution of Pearson correlations of the mean methylation value of high-quality CpGs across age bins is shown. CpGs with absolute correlations near 0.5 and 1 were selected (purple rectangles) for heatmap construction. **b** Heatmap showing methylation patterns for selected highly correlated and anti-correlated CpGs with age. The top four clusters were selected for enrichment analyses. **c**–**f** Network topology-based enrichment analysis was performed on genes associated with cluster 1 (**c**), cluster 2 (**d**), cluster 3 (**e**), and cluster 4 (**f**) CpGs. The top 15 categories with less than 1000 genes were selected using maximum weighted set cover and plotted as bar plots of the negative log of the FDR

We were also interested in what insights our clock could provide into the biology of aging. To see how CpG methylation values change with chronological age in our dataset, we binned our 8045 datasets into 15 chronological age bins roughly 5 years apart. We then calculated the average methylation values of our approximately 200,000 filtered CpGs and observed four peaks (Fig. 6a). Clustering the CpGs specific to the four peaks, we noticed that there were two types of behaviors captured: CpGs that gradually increase or decrease with increasing chronological age, and those that increase or decrease only at chronological ages > 90 (Fig. 6b).

Similarly to before [27], we used a network topology–based enrichment analysis [28] to identify gene ontology (GO) [29] terms enriched among genes associated with CpGs from each of those clusters. Biological processes associated with transcription, cell–cell signaling and hormone signaling, cell-cycle, and protein metabolism were significantly enriched among genes whose methylation gradually increases with age (Fig. 6c). Genes whose methylation dramatically jumps at very old age were enriched for cell-cycle, DNA damage response, autophagy, organelle assembly and localization, and viral process (Fig. 6d). Processes enriched among genes associated with CpGs that decrease methylation gradually with chronological age include protein metabolic processes and hemostasis, and synapse/vesicle signaling (Fig. 6e). Finally, terms enriched among genes associated with CpGs that lost their methylation dramatically at old chronological age include cell cycle, surface receptor cell–cell signaling, cellular component organization, and peptidyl-threonine modification (Fig. 6f).

We additionally explored how CpG variability changed with chronological age and delta CheekAge. We began by taking the same cohorts of samples binned into 5-year age bins and calculated the variance of the approximately 200,000 CpGs among samples in each of those age bins and then correlated the CpG variance with chronological age. We noticed a general increase in CpG variance with chronological age (Fig. S13a) and clustered the variance of CpGs with absolute correlation greater than 0.5 into three main clusters (Fig. S13b). We then took the genes associated with the CpGs in each of the top clusters and calculated enrichment of GO terms using a network topology–based enrichment approach. The largest cluster, which included CpGs that peaked in variance around the late 70s and mid-80s age ranges, was significantly enriched for terms associated with cornification, keratinization, intracellular signaling, cell cycle transition, and DNA-templated transcription (Fig. S13c). Similar to the first cluster, the second largest cluster containing CpGs that peaked in variance in the very late 80s and 90s age ranges was significantly associated with DNA-template transcription, intracellular receptor signaling, cell-cycle processes, and transcription (Fig. S13d). The final cluster contained CpGs that decreased in variance with chronological age and were associated with mRNA processes, splicing, cornification, intracellular signaling, and development (Fig. S13e).

We wondered if the top-weighted clusters among the 100 models comprising our clock were enriched for specific biological processes or terms. We started by selecting the CpG clusters with absolute averaged weights greater than two, which represented the top 1.33% of all CpG clusters (Fig. S14a). The top negative weighted model clusters were significantly associated with regulation of stress-activated MAPK cascade, platelet-derived growth factor signaling, and mitochondrion organization (Fig. S14b). Meanwhile, the top positive weighted model clusters were associated with various developmental and differentiation pathways, especially of the nervous system, as well as autophagy, protein localization, and cell–cell signaling (Fig. S14c).

We next explored the genomic annotation enrichment of CpGs. Taking the genomic annotations of the entire EPIC array as background, we calculated the enrichment of four sets of CpGs among CpG islands, CpG shores, CpG shelves, and Open Sea genomic elements (Fig.S14d). Overall, we noticed an enrichment of CpGs among CpG Islands, CpG shores, and CpG shelves, and a corresponding depletion of CpGs among Open Sea elements for the approximately 200,000 CpGs used to predict CheekAge, the CpGs corresponding CpG clusters with absolute weights > 2, the top 100 age-correlated CpGs, and CpGs from the top 100 age-correlated CpG clusters.

We then asked whether there was GO term enrichment among genes associated with the CpGs that were most correlated with delta CheekAge. We started by taking the CpGs with delta age correlation greater than 0.2 (0.38%) or less than − 0.2 (0.14%), of the approximately 200,000 CpGs used for clock training (Fig. S15a). We found that DNA-binding transcription factor activity, neuronal development and synaptic transmission, IkB/NFkB signaling, and post-transcriptional regulation were significantly enriched among genes associated with CpGs negatively correlated with delta CheekAge (Fig. S15b). Meanwhile, nervous system development, cell–cell signaling, cell death, and MAPK cascade were significantly enriched among genes associated with CpGs positively correlated with delta CheekAge (Fig. S15c).

Next, differentially variable CpGs were identified between cohorts with relatively low (< − 5 years, representing 8.5%) and relatively high (> 5 years, representing 9%) delta age values (Fig. S15d). GO terms significantly enriched among genes associated with CpGs that are more variable for lower delta CheekAges included cell cycle and DNA damage terms, protein localization to organelles, RNA processing, and calcium ion transport (Fig. S15e). GO terms significantly enriched among genes associated with CpGs that are more variable in samples with higher delta ages involved developmental terms (Fig. S15f). The full details of the enrichment results described in this section are tabulated in Supplementary Table 14.

To explore the relationship between CpGs and lifestyle and health factors, we calculated the correlations between all of our approximately 200,000 high-quality CpGs with lifestyle/health factors, chronological age, sex, race/ethnicity, and predicted cell type compositions (Fig. S16a). We then took the top 100 most correlated or anticorrelated CpGs associated with each of the 18 factors considered (Table S15) and calculated the overlap between them to see if each factor was associated with unique or shared CpGs (Fig.S16b). We found that cell-type proportions, alcohol, smoking, race/ethnicity, and exercise typically correlated with unique sets of top 100 CpGs, while CpGs associated with chronological age, stress, social satisfaction, education, immune health, and self-perceived aging were typically shared with other factors. As with the individual CpGs, we determined the correlation of the CpG clusters that were used as inputs for CheekAge clock construction with the 18 lifestyle/health, cell-type, and demographic factors (Fig. S17a). We then took the 100 most correlated CpG clusters for each of the 18 factors (Table S16) and plotted the overlap between them (Fig. S17b). As with the individual CpGs, cell type, race/ethnicity, smoking, and exercise tended to correlate with unique top clusters, although typically a higher proportion of the top clusters were shared among at least two factors. Chronological age, percentage of diet that is plant-based, stress level, social satisfaction, education level, immune health, self-rated health, self-perceived aging, and sleep quality tended to correlate with CpG clusters that were common to more than one lifestyle or health factor.

### Building an interactive web application for data exploration and CheekAge prediction

To help facilitate data exploration and analysis, and for others to be able to use the CheekAge clock, we created an interactive web portal called CheekAge Explorer. This tool allows users to explore the CpG methylation values and survey responses using interactive plots. Users are also able to generate plots of any variable as a function of a second. Continuous variables are shown as scatterplots with best-fit lines, categorical data (e.g., exercise activity) can be combined with continuous data using boxplots, and two categorical variables can be explored using mosaic plots [30]. Next, users can specify CpGs using gene symbols or CpG cg IDs and plot the change as a function of various factors. Finally, if users have the beta or *M*-values from EPIC V1 or V2 arrays, CheekAge Explorer can be used to calculate CheekAge. The app is available free of charge for academic use at http://cheekage.tallyhealth.com/.

## Discussion

In the course of developing this next-generation epigenetic aging clock optimized for adult buccal tissue, we noted several interesting observations. For example, several lifestyle and health factors were associated with chronological age in both sexes. In male and female subjects, chronological age initially decreased with increased self-rated health at the low end of the scale. From the middle to the higher end of increasing self-rated health, the associated chronological age increased significantly. Stress levels decreased and immune health increased with chronological age in male and female subjects, as did the percentage of a diet that was plant-based. The observation that chronologically older subjects perceived themselves as being healthier, feeling less stressed, having increased immune health, and consuming less meat could be the consequence of bias in study participation. Among older prospective subjects, healthier people may have been more interested in participating in this research. Alternatively, there could be a bias in survivorship, wherein subjects who feel less healthy, more stressed, have decreased immune health, and consume more meat have an increased rate of early mortality and fewer of those subjects survive into old age. Survivorship bias has, for example, been reported to diminish the observable relationship between age-related macular degeneration risk and smoking [31].

There were also significant differences in how chronological age was associated with smoking and BMI. Not smoking was more strongly associated with chronological age in male subjects and increased BMI moderately associated with higher chronological age in females. In contrast, increased BMI was more strongly associated with lower chronological age in males. The weaker association between increased BMI and increased chronological age in females could be due to sex-based differences in adipose tissue phenotypes [32]. The stronger association between increased BMI and decreased chronological age in males could be again due to survivorship bias, where males with increased BMI are at greater risk of mortality and, therefore, less likely to be in the older cohort [33]. Smoking and BMI were also among the factors most significantly contributing to delta age when delta age was modeled as a linear combination of survey factors. The most significant factor was race/ethnicity, which may be because non-white participants in our beta cohort were significantly less likely to drink and smoke. The five most significant lifestyle/health factors in descending order were BMI, smoking, alcohol, social satisfaction, and stress level, all of which are relevant to health and known to be associated with mortality risk.

One finding of interest was that several methylomic clusters exhibited more variability with age. These findings corroborate a report by Slieker et al., which reported that aging is synonymous with an increase in methylomic variation [34]. According to our data, variance for distinct clusters appears to ramp up around 70 years of age. Related work from the laboratory of Dr. Tony Wyss-Coray has shown that the human plasma proteome undulates with age, with noticeable peaks of differential expression occurring at ages 34, 60, and 78 [35]. A previous meta-analysis in human transcriptomic data analogously identified a plethora of genes that were both variably and differentially expressed after age 70 [36]. While multi-omics data from the same individuals is ultimately needed to better understand how the molecular landscape becomes aberrant with age, current evidence suggests that multiple molecular systems are dysregulated in the final decades of life.

One curious finding was that a higher delta age was significantly associated with SARS-CoV-2 and non-SARS-CoV-2 respiratory infections. While the data is mixed [37], multiple reports have linked an active SARS-CoV-2 infection to an elevated epigenetic age [38, 39]. The ability of an infection to transiently impact a biomarker is not particularly surprising. For instance, an active SARS-CoV-2 infection has been connected to a reduction in grip strength [40], a higher level of C-reactive protein [41], and an increased amount of GDF15 [42]. One possible explanation for our finding is that a subset of the CpGs used in our clock are annotated to immune system genes and may be sensitive to inflammation. Indeed, one of our enrichment analyses looked at different clusters that gained or lost methylation with age. In cluster 2, which becomes hypermethylated with age, one of the top terms was “viral process.” In a separate enrichment analysis looking at clusters with a delta age correlation less than − 0.2, “l-kappaB kinase/NF-kappaB signaling” was the fourth most enriched term. The dysregulation of the immune system with age in vertebrate animals is well characterized. For example, a previous epigenetic and transcriptomic analysis found that innate immune pathways are commonly dysregulated across African turquoise killifish, rats, and humans [43]. Plasma proteins associated with both the adaptive and innate immune systems are also uniquely adept at predicting age in humans [44].

Additional research is warranted to better understand how our CheekAge clock behaves over time and to uncover novel factors linked to a younger or older epigenetic age. Future investigations should also examine whether health-promoting interventions—such as adopting a Mediterranean diet, increasing weekly resistance-training, or cutting out ultra-processed food—can significantly decrease CheekAge in a randomized clinical trial setting. Since aging clocks and machine learning models are highly complex, it would also be fruitful to gain a deeper understanding of the CpG sites and CpG clusters used by our ensemble model, including their associated biology. In addition to utilizing DNA methylation, lifestyle information, and health information, future models that use artificial intelligence to incorporate additional measurement modalities may unlock greater accuracy, reliability, and utility.

### Supplementary Information

Below is the link to the electronic supplementary material.

Supplementary Figure 1 (TIF 13156 KB)

Supplementary Figure 2 (TIF 19367 KB)

Supplementary Figure 3 (TIF 7812 KB)

Supplementary Figure 4 (TIF 11712 KB)

Supplementary Figure 5 (TIF 10124 KB)

Supplementary Figure 6 (TIF 19478 KB)

Supplementary Figure 7 (TIF 10426 KB)

Supplementary Figure 8 (TIF 16112 KB)

Supplementary Figure 9 (TIF 11514 KB)

Supplementary Figure 10 (TIF 21429 KB)

Supplementary Figure 11 (TIF 6135 KB)

Supplementary Figure 12 (TIF 10457 KB)

Supplementary Figure 13 (TIF 9682 KB)

Supplementary Figure 14 (TIF 8143 KB)

Supplementary Figure 15 (TIF 16618 KB)

Supplementary Figure 16 (TIF 16643 KB)

Supplementary Figure 17 (TIF 16330 KB)

Supplementary Methods (DOCX 102 KB)

Supplementary Table 1 (XLSX 72 KB)

Supplementary Table 2 (XLSX 1606 KB)

Supplementary Table 3 (XLSX 129 KB)

Supplementary Table 4 (XLSX 128 KB)

Supplementary Table 5 (XLSX 75 KB)

Supplementary Table 6 (XLSX 129 KB)

Supplementary Table 7 (XLSX 80 KB)

Supplementary Table 8 (XLSX 128 KB)

Supplementary Table 9 (XLSX 83 KB)

Supplementary Table 10 (XLSX 612 KB)

Supplementary Table 11 (XLSX 1046 KB)

Supplementary Table 12 (XLSX 74 KB)

Supplementary Table 13 (XLSX 90 KB)

Supplementary Table 14 (XLSX 2219 KB)

Supplementary Table 15 (XLSX 137 KB)

Supplementary Table 16 (XLSX 233 KB)

## Data Availability

The raw data are proprietary and cannot be disclosed. To allow the public to mine our methylomic data and predict CheekAge using their own methylomic datasets, we have created a free-to-use ShinyApp at http://cheekage.tallyhealth.com/, which is provided for academic use. Data uploaded to the app is not stored and deleted after use. The Gene Expression Omnibus identifiers for the publicly available methylomic datasets used in this paper are as follows: GSE111165, GSE167202, GSE151617, GSE197674, GSE183647, GSE179847, GSE216024, GSE214901, GSE199057, GSE188593, and GSE172365.

## Code availability

The underlying code for this study is not publicly available but may be made available to qualified researchers upon reasonable request from the corresponding authors. Please see the Supplemental Methods for a list of functions and libraries used.
