# Supplementary material for: CheekAge: a next-generation buccal epigenetic aging clock associated with lifestyle and health
Source: GeroScience. 2024 Mar 5;46(3):3429–43. doi: 10.1007/s11357-024-01094-3 (PMC11009193; doi:10.1007/s11357-024-01094-3)
Supplement: Supplementary file 18 — Supplementary Methods (DOCX 102 KB) [file 11357_2024_1094_MOESM18_ESM.docx]

*EPIC preprocessing*

Computational processing of data was carried out using the R programming language version 4.2.3 (https://www.r-project.org/). The raw idat files were preprocessed using the minfi (v 1.44.0) preprocessing pipeline^1^, starting from ~850,000 CpGs. In short, datasets were read in using the read.metharray.exp function, Noob normalization^2^ was applied, and low quality CpGs, calculated with the detectionP function, were filtered out by removing CpGs whose p-values were above 0.05 in more than 5% of the samples measured. Then, cross-reactive loci were removed using the dropXreactiveLoci (https://github.com/markgene/maxprobes) and loci with known SNPs using the dropLociWithSnps function included with minfi. Next, the getQC function was used to remove samples with average median methylated and unmethylated signals (meds) less than 9.5. Sex was predicted using the minfi getSex function using the beta values, which uses the intensity across the sex chromosomes to classify samples. Cell type prediction was carried out using the EpiDISH package (v 2.14.1)^3^, using the centEPiFibIC and centBloodSub references and RPC method with maxit = 100000 to estimate cell proportions. Epithelial, neutrophil, and the sum of B cells, natural killer cells, CD4-T cells, CD8-T cells, monocytes, and eosinophils (collectively called otherImmune) were calculated. Next, we determined CpGs that significantly correlated with sex, array ID, slide ID, or meds, and CpGs with correlation significance q-values less than 1e-15 for any of those factors were filtered out to avoid including CpGs associated with technical variables. Finally, CpGs that had a beta value variance greater than 0.005 among 380 biological replicate samples were removed. These filtering steps resulted in approximately 200,000 high-quality CpG sites. Beta values were used to calculate M values according to:

$M_{i}=log_{2}(\frac{beta_{i}}{1-beta_{i}})$,

where $M_{i}$ is the ith M value, and $beta_{i}$ is the ith beta value bound to be between 0.00001, and 0.99999 to avoid infinities. Unless otherwise stated, M-values were used for training and subsequent analyses and were selected over beta values to help improve sensitivity to small changes near the extreme values.

*CheekAge clock: preprocessing*

We used a linear model to represent the predicted age as a function of inputs:

$$Age=w_{0}+\sum_{i}^{n} w_{i}x_{i}$$

where the predicted age, $Age$, is a function of an offset $w_{0}$, and the sum of $n$ inputs $x$ that are multiplied by weights $w$. Instead of using the CpG methylation values as inputs directly, we combined multiple CpG inputs into clusters of CpG averages by first calculating the principal components (PCs) of the scaled and centered input M values using the *prcomp* R function, followed by hierarchical clustering using function *hclust* with the distance defined as 1 - correlation of the first 25 PCs of the 8,045 samples and ward.d2 agglomeration to identify the top 10,000 clusters of CpGs with similar PC component correlations. The CpG methylation values in each cluster were averaged, resulting in 10,000 biomarkers. This is similar to recent methods that use combinations of CpGs as inputs for age prediction ^4,5^. In addition to the top 10,000 CpG clusters, we added the predicted sex, epithelial, neutrophil, and other immune cell proportions as inputs to model training.

*Constructing a first-generation clock*

We used the caret (v 6.0-94) R package^6^ to train a glmnet (v 4.1-7) penalized linear regression model^7^ using the preprocessed CpG cluster inputs along with predicted sex and epithelial, neutrophil, and other immune cell proportions, to predict chronological age only. We used a 10-fold cross validation approach that was repeated five times to select the hyperparameters with the lowest root mean square error (RMSE) and with predicted age values limited to the 10-110 year range. The final model was then trained on the full dataset using the optimal hyperparameters of alpha = 0.1 and lambda = 1.07.

*Principal component analysis of CheekAge input clusters*

To explore if lifestyle and age were encoded in the methylation cluster data, we ran a principal component analysis on the entire training data with the *prcomp* R function decomposing the data into 10,000 PCs and used the *cor.test* R function to calculate the significance of the correlation between lifestyle/health, technical, and demographic variables with the first 18 PCs. The resulting negative log of the significance of correlation of variables with PCs were clustered using the *heatmap* R function with the default Euclidean distance function and complete agglomeration method.

*CheekAge clock: construction*

To build a clock sensitive to lifestyle and health factors, we needed to optimize for both the accuracy of the predicted age as well as the correlation of the residuals of those predictions with survey responses. To enable this type of nested multi-variable optimization, we used a simulated annealing optimization strategy^8^ with the minimization function:

$$score_{s}=k_{1}RMSE^{2}+k_{2}RMSE+k_{3}\sum_{f} (-log(c_{f}))+k_{4}r+k_{5}l$$

where scores is the score at iteration step $s$, RMSE is the root mean squared error of the predicted ages compared to the chronological ages, $c_{f}$are the p-values of the correlation significance of the 12 survey factors with the age residuals for the 12 factors $f$ calculated using the cor.test R function for the Pearson correlation, $r$ is the sum of the model weights squared (L2 regularization), and $l$ is the sum of the absolute model weights (L1 regularization)^9^. To ensure that no one factor correlation dominated the optimization, we capped the $-log(c_{f})$ at -7 for each factor. The constants $k_{1}$, $k_{2}$, $k_{3}$, $k_{4}$ and$k_{5}$, were manually tuned to be 15, 100, 130/12, 0.2, and 0.2, respectively. We found that these values balanced the optimization toward both accuracy and very high correlation with lifestyle and health factors, while minimizing model complexity (Fig. S4).

Initially model weights were set to 0 and the model intercept was set to the average of the chronological ages of all samples. Then optimization proceeded for several steps using the simulated annealing algorithm with specific modifications described below. Each optimization step consisted of several parts. First, weights were randomly changed by adding values between -0.04*Temp and 0.04*Temp in addition to a value between -0.01 and 0.01, where Temp is the current annealing temperature. The new weights were then used to calculate new ages for all samples and the current score was calculated according to the equation above. If the score was lower than the previous score, the weights were kept, otherwise, they were kept with a probability equal to $e^{(score_{s-1}-score_{s})/(2Temp^{2})},$allowing for higher score solution spaces to be sampled, which is important for avoiding the optimization getting trapped in local minima during the annealing process while Temp was still relatively high. At the end of each step the annealing temperature, Temp, was multiplied by a factor of *0.99995 / x^round-1^*, where round is the annealing round (1 to 10), and *x = sqrt(1.00004)*, such that the cooling rate starts at 0.99995 for the first round, and decreases to 0.99991 for the 10th round. The annealing temperature was set to 1 at the beginning of each optimization round, and the round was concluded when the annealing temperature dropped to 0.01, which typically meant anywhere from 100,000 to 200,000 iterations per round. Finally, to avoid premature termination, every 1,000 iterations the total number of global improvements was used to reheat the system by multiplying the Temp by $1.005^{improvements}$, where $improvements$ is the number of times the global best score was decreased in the last 1,000 iterations. This reheating strategy allowed the system to reach a new more optimal score equilibrium even at the end of an optimization round by keeping the temperature relatively high while many favorable changes were being made (e.g., compare the orange curves to the purple curves in Supplementary Figure 4c).

To further minimize model complexity and improve generalizability, the number of inputs was decreased with each optimization round. At the end of each round, the inputs with the smallest 23% of absolute inputs were removed, and the optimization was restarted. This meant that at the end of the 10th round, only 1,000 top inputs out of the 10,004 starting inputs were kept, resulting in much simpler final clock models with comparable accuracy and factor correlations (Fig. S4b,c). Importantly, since our survey results were prone toward healthier responses for chronologically older volunteers (Fig. S1e), we trained the clock using the residuals of the best-fit linear regression instead of the difference between predicted and chronological age. This was important to avoid trivial local minima during model optimization involving a decrease in the overall slope of the fit, which artificially increased delta CheekAge values for younger and decreased delta CheekAge values in older aged samples. However, the final model fits still tended to produce somewhat higher delta ages for younger individuals and lower delta ages for older individuals due to the overrepresentation of middle-aged volunteers in our cohort (Fig. S1a). Therefore, model weights were rotated (see below) to ensure predictions were centered on the diagonal.

*CheekAge clock: ensembling*

The 10-round optimization was repeated 1,098 times, resulting in 1,098 unique models being trained on the same data. However, since the optimization was stochastic, the final score was different for all trained models, and models arrived at different optimal solutions (Fig. S4e). Therefore, we experimented with averaging the predictions of multiple models using a weighted mean. Specifically, the weights, W, of all 1,098 models m were calculated as:

$W_{m}=(1000/score_{m})^{k}$,

where $score_{m}$is the final score for model m, and the power, k, was set to 4 to further bias the clock predictions toward the most optimized models. The ensemble mean age prediction, CheekAge, was then calculated as:

$$CheekAge=\frac{\sum_{m} W_{m}CheekAge_{m}}{\sum_{m} W_{m}}$$

We showed that an ensemble containing the weighted average of 100 models with the smallest scores was sufficient (Fig. S5), so the 100 models with the lowest scores were combined into our final CheekAge ensemble.

*Rotating the clock weights or predictions*

Aging clocks frequently show chronological age bias, which result in under- or over-prediction for specific segments of the chronological age distribution and can confound analyses made using delta age^10^. To ensure that clock predictions were evenly distributed about the diagonal, we fit a linear approximation to the predicted ages as a function of chronological ages using the lm function in R. We then applied a correction to the model weights as follows:

$w_{0}={(w}_{0}-b)/m$,

$w_{i>0}=w_{i}/m$,

where $b$ is the best fit linear intercept, and $m$ is the best fit linear slope. This adjustment could similarly be applied directly to the prediction values by adjusting each predicted age according to:

${Age}_{rotated}={(Age}_{unrotated}-b)/m$.

Similar methods have been used in the past to adjust a proteomic clock^11^, an image-based clock^12^, and an epigenetic clock^13^.

*Calculating correlation of survey factors with age and delta age*

To evaluate if age or delta age was significantly correlated with lifestyle and health factors, we modeled the chronological age or delta age as a linear function of survey factors, demographic factors, and technical factors using the *lm* R function:

$$age\sim E+N+OI+BMI+hf+af+st+sl+im+ed+so+di+ex+sex+sm+al+race,$$

$$delta age\sim age+E+N+OI+BMI+hf+af+st+sl+im+ed+so+di+ex+sex+sm+al+race,$$

where $delta age$ is the predicted age minus the chronological age, $age$ is the chronological age, $E$ is the epithelial cell proportion, $N$ is the neutrophil cell proportion, $OI$ is the other immune cell proportion, $BMI$ is the calculated body mass index, $hf$ is self-rated health, $af$ is self-perceived aging, $st$ is stress level, $sl$ is sleep quality, $im$ is relative immune health, $ed$ is education level, $so$ is social satisfaction, $di$ is the fraction of a diet that’s plant-based, $ex$ is weekly exercise, $sex$ is the predicted sex, $sm$ is smoking status, $al$ is alcohol habits, and $race$ is race/ethnicity. For more factor details, please see Supplementary Figure 1 and Supplementary Tables 2, 3, and 7. Significance of the linear coefficients are calculated using the t-values generated by the *summary.lm* R function. Importantly, the scaling for these variables is different, Cell type proportions sum to 1, lifestyle/health factors range between 0 and 1, and age and BMI are not scaled.

Importantly, during model training we used independent tests for significance for each lifestyle/health factor and capped the log significance at -7 instead of fitting a multivariate linear regression to all variables (Figs. S4 and S5). This was done to significantly speed up the optimization step during model training, but the full linear model was used to evaluate the significance of the linear association while accounting for other factors.

*Exploring an ensemble of 100 clocks*

As described above, the CheekAge clock is a collection of 100 independently trained linear models. Each model consists of 1,000 inputs that were selected from the 10,004 inputs. To better understand the differences and similarities of these models, we ran several analyses. First, we directly compared the number of inputs that appeared in 1 to 100 models (Fig. S4e). Next, we used the squared Euclidean distance between the weights of any two models $a$ and $b$ as the distance function:

$dist_{a,b}=\sum_{i} \left( w_{a,i}-w_{b,i} \right)^{2}$.

and generated a clustered heatmap of the clocks using the 9,952 CpG cluster inputs that were utilized by at least one model. The *heatmap* R function was used with agglomeration function ward.d2, and the CpG clusters were sorted by their mean weight across all 100 models (Fig. S6a). Similarly, we used the 100 models to generate predictions for the entire set of 8,045 samples and used the squared Euclidean distance of the predictions across the 8,045 samples as a distance measure:

$dist_{a,b}=\sum_{i} \left( {\Delta CheekAge}_{a,i}-{\Delta CheekAge}_{b,i} \right)^{2}$,

where ${\Delta CheekAge}_{a,i}$, and ${\Delta CheekAge}_{b,i}$ are the delta ages for model $a$, and model $b$ for the sample $i$, respectively Fig. S6b). Finally, to better understand how lifestyle/health, demographic, and technical variables were encoded for the 100 models, we started by fitting a multivariate linear model of delta age as a function of the 18 variables described above. We then calculated the significance of the coefficients using the *summary.lm* R function. Next, we calculated the negative log of those significances and performed hierarchical clustering using the function *heatmap* with ward.d2 agglomeration and distance measure:

$dist_{a,b}= 1-cor(C_{a}{,C}_{a})$,

where $C_{a}$ and $C_{b}$ are the vectors of negative log p-values of the correlations of lifestyle, demographic, and technical variables with delta age predicted by models a, and b, respectively (Fig. S6c).

*Clock metric calculations*

Delta age was defined as the predicted CheekAge, $Age_{p}$, minus the chronological age, $Age_{c}$. We used five main metrics to evaluate the quality of a clock for a given set of n pairs of predicted and chronological ages. RMSE is calculated as:

$$RMSE=\sqrt{\frac{\sum_{i}^{n} (Age_{i,c}-Age_{i,p})^{2}}{n}}$$

Mean absolute error (MAE) is calculated as:

$MAE= \frac{\sum_{i}^{n} \left| Age_{i,c}-Age_{i,p} \right|}{n}$.

The squared R is calculated as:

$$R^{2}=\frac{\left( \sum_{i}^{n} (Age_{i,c}-\underline{Age_{i,c}})(Age_{i,p}-\underline{Age_{i,p}}) \right)^{2}}{\sum_{i}^{n} {(Age_{i,c}-\underline{Age_{i,c}})}^{2}\sum_{i}^{n} {(Age_{i,p}-\underline{Age_{i,p}})}^{2}}.$$

The mean absolute bias (MAB) is calculated by first fitting a cubic spline to the paired data using the *gam* function in the mgcv package (v 1.8-42), using default parameters. Then the mean absolute residuals between the spline and chronological age are averaged:

$MAB=$ $\frac{\sum_{i}^{n} \left| Age_{i,s}-Age_{i,c} \right|}{n}$,

where $Age_{i,s}$ is the age predicted using the spline approximation to the predicted age as a function of chronological age. In effect MAB is an estimate of the non-linearity of the predicted age compared to the chronological age and higher values indicate deviations from the diagonal. Finally, to estimate the error between replicates, we calculate the mean replicate error (MRE):

$$MRE=\frac{1}{m}\sum_{r}^{m} \frac{1}{n_{r}}\sum_{i}^{n_{r}} \left| Age_{i=r,p}-\underline{Age_{i=r,p}} \right|$$

This metric is the mean replicate distance from the mean for all replicates across replicate sets, which consists of 190 pairs of samples with high yields from both collection vials. While this metric works for any number of replicates per sample, it reduces to the mean half distance between duplicates such that:

$MRE_{duplicates}=\frac{1}{2m}\sum_{r}^{m} \left| Age_{i=r1,p}-Age_{i=r2,p} \right|$,

where $Age_{i=r1,p}$ and $Age_{i=r2,p}$are the predicted ages of the first and second replicate for replicate pair $r$, respectively.

*Evaluating CheekAge in external, publicly available datasets*

First, to test our clock in an external buccal dataset, we downloaded and processed GSE111165 containing buccal, saliva, and blood datasets from 21 patients with intractable epilepsy^14^. We quantified the accuracy of CheekAge predictions in all three tissues (see *Clock metric calculations* above).

The second dataset (GSE167202) contained blood methylation data from individuals with SARS-CoV-2 infection (n = 164), individuals without SARS-CoV-2 infection (n = 296), and individuals with a different acute respiratory infections (n = 65) aged 17-96 years^15^. Delta age was modeled as a function of sex, race, chronological age, white blood cell count, and COVID status:

$\Delta CheekAge \sim sex + race + age + white blood cell count + infection status$.

We also identified a human skin dataset (GSE151617) which included both healthy controls and progeria samples as well as information about rapamycin treatment and ABT-263 treatment^16^. Altogether, there were 50 samples from progeria and non-progeria samples. We assessed whether delta age estimation was significantly associated with any of the variables and identified progeria status as significantly associated with delta age after controlling for other confounding variables:

$\Delta CheekAge \sim age+progeria+ABT+Rapamycin$.

A separate dataset (GSE197674) was part of a larger study focused on understanding accelerated aging in childhood cancer survivors and included chronological age, sex, and whether specific treatments were carried out^17^. The dataset was collected from the blood of 2,138 adult survivors of childhood cancers and processed using MethylationEPIC arrays. Delta age was modeled as a function of gender (provided in the external data), chronological age, and treatment (indicated by 0 or 1):

$\Delta CheekAge\sim gender+age+brainRT+chestRT+apRT+alk+anth+vinc+cort+plat+epip$,

where $brainRT$, $chestRT$, $apRT$, $alk$, $anth$, $vinc$, $cort$, $plat$, and $epip$ represent brain radiation therapy, chest radiation therapy, abdominal/pelvic radiation therapy, alkylating agents, anthracyclines, vincristine, corticosteroids, platinum, and epipodophyllotoxins, respectively.

We also tested a dataset (GSE183647) containing methylation data from meningioma tissues classified as grade 1 (benign, n = 388), grade 2 (atypical, n = 142), or grade 3 (malignant, n = 35)^18^. Tissue donors were aged 5-90 years. We downloaded the raw methylation data generated from an Infinium MethylationEPIC array. Delta age was modeled as a function of age, sex, and cancer grade.

$\Delta CheekAge \sim sex + age + cancer grade$.

Another dataset (GSE179847) contained methylation data from primary human fibroblasts run on an Infinium Methylation EPIC array^19^. We divided data obtained from patients with a normal genotype (n = 5) and only compared untreated samples (Controls). Cell lines from each sample were passaged up to 30 times and, for comparison, passages were combined into Early (1-10) (n = 51), Medium (11-20) (n = 49), and Late (21-30) (n = 18). Delta age was calculated for each passage of each sample of normal, untreated cells and modeled as a function of chronological age of donor, sex of donor, and number of days per passage:

$\Delta CheekAge \sim sex + age + number of days per passage + passage number category$ .

The next dataset (GSE216024) contained methylationEPIC data from 140 human rectal samples sampled across diverse chronological ages, races, and sexes^20^. We identified a significant linear relationship between delta age and BMI after controlling for age, race, sex, and adenoma status:

$\Delta CheekAge \sim age+sex+BMI+adenoma+race$.

We also tested for significant associations in three additional external datasets, which showed mixed or difficult to interpret results. We first looked at a colorectal cancer dataset (GSE199057), where we tested for an association of disease status, sex, age, and race:

$\Delta CheekAge \sim Status+Sex+Age+Race$.

Second, we looked at a dataset (GSE172365) derived from cultured epithelial cells antagonized with rhinovirus^21^. We tested for an association with age, sex, ethnicity, smoking status, rhinovirus treatment, steroid administration, asthma diagnosis, and DNA concentration:

$$\Delta CheekAge \sim Age + Sex + Ethnicity + Smoking + Treatment + Steroid + Asthma + DNAConc$$

Finally, we explored a melanocytic nevi dataset (GSE188593), where we checked for an association between delta CheekAge and age, tissue classification, and sex^22^:

 $\Delta CheekAge \sim Age+Classification+Sex$.

*Predicting age in our buccal data using external clocks*

We ran four existing methylation age clocks in our 8,045 volunteer dataset using a publicly available R package, MethylCIPHER^23^. We selected four recently-published clocks for which all CpGs were available in our dataset: PhenoAge^24^, Horvath 2018^25^, Zhang 2019^26^, and PedBE^27^. For each clock, we calculated clock performance metrics. Additionally, to correct the inherent chronological age bias of each clock, we rotated the predictions and recalculated the clock performance metrics.

*Understanding methylation mean and variance changes with age*

To understand how methylation mean and variance changes with chronological age, we binned samples into 15 bins of approximately five years from 20-90 years and calculated the mean and variance of each of the approximately 200,000 preprocessed CpG M values in each of the bins. Looking at the correlation of mean CpG methylation with chronological age, we selected just the CpGs with absolute correlations 0.9-1.0 and 0.45-0.55, since those were overrepresented in the population. We then created a clustered heatmap with the *heatmap* function using 1-correlation as the distance function, and ward.d2 agglomeration. The *cutree* R function was used to identify the top four clusters of CpGs changing with chronological age, which were subjected to enrichment analysis as described below. To gain insights into methylation variance changes with chronological age, we applied a similar strategy: CpGs whose variance had an absolute correlation higher than 0.5 with chronological age were selected for clustering. A clustered heatmap was created with those CpG variances using the *heatmap function*, 1-correlation as the distance function, and ward.d2 agglomeration. The top three clusters were identified with the *cutree* R function, and enrichment analysis was performed on each of the clusters.

*Exploring clock methylation patterns*

For each of the 10,000 CpG clusters used as input for model training, the average weights among the 100 models that comprise the CheekAge clock was calculated. The top clusters were selected as those with weight greater than 2 or less than -2, and all CpGs for clusters with high mean weights and low mean weights were compiled. Then all genes associated with the compiled CpGs were used as input for a network topology-based enrichment analysis. Similarly, the correlation of all approximately 200,000 high quality CpGs with delta age was calculated and CpGs that had correlations less than -0.2 or higher than 0.2 were set aside. Next, genes associated with each of those positively or negatively correlated CpGs were used as inputs for a network topology-based enrichment analysis. Finally, differentially variable CpGs were identified between cohorts with relatively low (< -5 year) and relatively high (> 5 year) delta ages using the *varFit* function of the *missMethyl* package (v1.30.0)^28^. Individuals with a delta age >5 years were classified as “positive” while individuals with delta age < -5 were classified as “negative”. This metric was named “direction” in the following input model for the analysis design, where the variables are the same as in section “Calculating correlation of survey factors with age and delta age” above:

$$Direction\sim age+E+N+OI+BMI+hf+af+st+sl+im+ed+so+di+ex+sex+sm+al+race$$

CpGs whose variance was significantly different between the high and low delta age groups were used to run enrichment analyses.

*Exploring the overlap of CpG and CpG cluster correlation with 18 factors*

To better understand how lifestyle/health, demographic, and technical factors are correlated with specific CpGs and CpG clusters, we started by calculating the Pearson correlation of all CpGs or CpG clusters used to build the CheekAge clock with chronological age, sex, race/ethnicity, alcohol, smoking, plant-based diet, exercise, stress levels, social satisfaction scores, education level, immune health, BMI, self-rated health, self-perceived aging, sleep quality, and predicted proportion of epithelial, neutrophil, or other immune cells. We then took the top 100 correlated CpGs or CpG clusters with each factor (Supplementary Tables 14 and 15) and plotted the overlap using the UpsetR R package (v. 1.4.0). The resulting upset plots show the number of CpGs or CpG clusters in each set and the membership across the original sets of top correlated CpGs or clusters for each factor. A maximum of 70 sets were shown and ordered by set size.

*GO enrichment analysis*

Enrichment analysis helps identify known biological processes overrepresented among input data. Similarly to before^29^, we used the *WebGestalt* tool^30^ to perform network topology-based enrichment analysis with the protein-protein interaction database BioGRID^31^ to identify Gene Ontology (GO)^32^ terms with significant overrepresentation. When the number of genes was greater than 500, we used the network retrieval & prioritization algorithm with default settings, which uses a random walk along the network to identify a subnetwork of genes for enrichment analysis (Figs. S13, S14, and S15e,f). When the number of genes was less than 500, we used the network expansion algorithm instead, with the number of top ranking neighbors set to ½ the number of input genes (Fig. S15c,d). Since gene symbols were used as inputs, we first identified the union of all genes annotated to the CpGs. When more than one gene was associated with a CpG, all were included. The final list of genes was then used as input for enrichment analysis. A false discovery rate (FDR) cutoff of 0.05 was set and terms with fewer than 1,000 genes, which represent more specific terms, were kept. The weighted set cover algorithm was used to identify up to 15 top sets of significant non-redundant GO terms using the *weightedSetCover* function in the WebGestaltR (v 0.4.5) R package. The negative log of the FDR was used to plot the significance of the enrichment. Importantly, since a partial overlap may still be significant, it is important to manually check the specific genes found to be overlapping with a term of interest. For a full set of terms identified, the number of genes in each term, the overlap, the enrichment ratio, and the specific genes please see Supplementary Table 14.

*Genomic enrichment analysis*

To identify the enrichment of a set of CpGs among genomic features, we used the Illumina EPIC annotation available through Bioconductor (https://doi.org/doi:10.18129/B9.bioc.IlluminaHumanMethylationEPICanno.ilm10b4.hg19), which includes CpG Island annotations for each CpG on the EPIC array. We combined the left and right shelves and left and right shore annotations for simplicity and used the full EPIC array annotation to determine the fold enrichment of CpGs among CpG Islands, CpG shores, CpG shelves, and Open Sea genomic features. We present the fold enrichment over the EPIC array background for four sets of CpGs of interest: the approximately 200,000 CpGs used for CheekAge prediction, the CpGs from CpG clusters with absolute model weights greater than 2, the top 100 CpG with the highest absolute pearson correlation to chronological age, and the CpGs from the top 100 CpG clusters with the highest absolute pearson correlation to chronological age.

*Interactive Shiny app*

To facilitate the exploration and analysis of our dataset and CheekAge clock, we constructed a free-to-use interactive web application using the shiny (v 1.7.4) R package (https://rdrr.io/cran/shiny/) as well as the plotly (v 4.10.1) R package (https://plotly-r.com/). The tool includes functionality for plotting the lifestyle and health questionnaire, demographic information, and prediction results for the full 8,045 dataset. It also provides a way of plotting the methylation status of all CpGs and a module for predicting CheekAge from an uploaded table containing beta or M values. The application is hosted on a dedicated cloud server at http://cheekage.tallyhealth.com/ and provided free of charge for academic use. Data is not collected or stored after use.

*Functions and arguments used*

Below is a tabulated version of the key functions and important arguments used to run analyses along with a brief description.

| **Function** | **Package** | **Arguments** | **Analysis** |
| --- | --- | --- | --- |
| preprocessNoob | minfi | default | Normalization |
| detectionP | minfi | default | Identifying low quality probes |
| getQC | minfi | default | Calculating median intensities |
| dropXreactiveLoci | maxprobes | default | Remove cross-reactive CpGs |
| dropLociWithSnps | minfi | default | Remove CpGs with known SNPs |
| getSex | minfi | default | Estimate sex |
| hepidish | EpiDISH | Ref1.m = centEPiFibIC.m, ref2.m = centBloodSub.m  h.CT.idx=3, method= RPC, maxit = 100000 | estimate cell type proportions |
| dmpFinder | minfi | type=”categorical” for categorial variables, type=”continuous” for continuous variables | For identifying CpGs that significantly associate with variables of interest |
| fast.prcomp | gmodels | scale=TRUE, center=TRUE | PCA |
| hclust | fastCluster | method=”ward.D2” | Clustering CpGs |
| lm | stats | See supplemental tables and methods for specific model design used | Identifying significant associations |
| vioplot | vioplot | default | Violin plots |
| train | caret | Age~., method=”glmnet” tuneLength=5, importance=TRUE | Gen 1 clock training |
| trainControl | caret | method=”repeatedcv”, repeats=5, number=10, selectionFunction=”best”, predictionBounds=c(10,110) | Gen 1 clock training |
| getGEO | geoQuery | filename= series_matrix.txt file | Processing public datasets |
| varFit | missMethyl | Matrix and model design are provided as arguments | Differential variance analysis |
| weightedSetCover | WebGestaltR | costs=1/-log(FDR),topN=15 | Enrichment analysis summary |
| heatmap | stats | Graphical parameters specific to individual heatmaps | Heatmap visualization |
| cor | stats | default | Correlation analyses |
| pairs | graphics | Upper.panel = panel.cor, diag.panel=panel.hist | Scatterplot matrices plot |
| upset | UpSetR | fromList, nsets=18, nintersects = 70, order.by = “freq” | Upset plots |
| boxplot | graphics | default | Box plots |
| hist | graphics | default | Histograms |
| predictAge | custom | The ensemble of models and the 10,000 clustered CpGs are input | Outputs a weighted mean of age predictions for each set of 10,000 CpG clusters by calculating an age for each model using model score as the normalization weight for each model |
| rotate | custom | The slope and intercept | For the model intercept, subtracts the intercept and divides by the mean, for all weights, divides by the mean |
| getM | custom | betas | log(betas/(1-betas),2) |
| RMSE | custom | x,y | sqrt(mean((x-y)^2)) |
| MAE | custom | x,y | mean(abs(x-y)) |
| bias | Custom + mgcv | x,y | Uses the gam function to fit a spline ot the data, then returns the mean absolute difference between the spline and the x |
| MRE | custom | Rep, age | mean(abs(age[rep==i&!is.na(rep)]-mean(age[rep==i&!is.na(rep)])). Returns a list of all differences between replicates and their respective mean predictions |
| SA | custom | CpGClusters, rep, num=1000 maxRound=10 | Runs simulated annealing optimization and returns the rotated optimized model. See methods for additional details |
| getCpGClusters | custom | M-value array, names of CpGs in clusters | For each cluster, average the M-values for the CpGs in the cluster and return the averages for all clusters and all samples. To be used for simulated annealing training, and predicting age given an ensemble of models |
| p.adjust | stats | method=”BH” | Multiple testing adjustment |

**Supplementary Methods References**

1. Aryee MJ, Jaffe AE, Corrada-Bravo H, Ladd-Acosta C, Feinberg AP, Hansen KD, Irizarry RA. Minfi: a flexible and comprehensive Bioconductor package for the analysis of Infinium DNA methylation microarrays. *Bioinformatics*. 2014;30:1363-1369. doi: 10.1093/bioinformatics/btu049

2. Fortin JP, Triche TJ, Jr., Hansen KD. Preprocessing, normalization and integration of the Illumina HumanMethylationEPIC array with minfi. *Bioinformatics*. 2017;33:558-560. doi: 10.1093/bioinformatics/btw691

3. Zheng SC, Breeze CE, Beck S, Dong D, Zhu T, Ma L, Ye W, Zhang G, Teschendorff AE. EpiDISH web server: Epigenetic Dissection of Intra-Sample-Heterogeneity with online GUI. *Bioinformatics*. 2019;36:1950-1951. doi: 10.1093/bioinformatics/btz833

4. Bernabeu E, McCartney DL, Gadd DA, Hillary RF, Lu AT, Murphy L, Wrobel N, Campbell A, Harris SE, Liewald D, et al. Refining epigenetic prediction of chronological and biological age. *Genome Med*. 2023;15:12. doi: 10.1186/s13073-023-01161-y

5. Higgins-Chen AT, Thrush KL, Wang Y, Minteer CJ, Kuo PL, Wang M, Niimi P, Sturm G, Lin J, Moore AZ, et al. A computational solution for bolstering reliability of epigenetic clocks: Implications for clinical trials and longitudinal tracking. *Nat Aging*. 2022;2:644-661. doi: 10.1038/s43587-022-00248-2

6. Kuhn M. Building Predictive Models in R Using the caret Package. *Journal of Statistical Software*. 2008;28:1 - 26. doi: 10.18637/jss.v028.i05

7. Friedman J, Hastie T, Tibshirani R. Regularization Paths for Generalized Linear Models via Coordinate Descent. *J Stat Softw*. 2010;33:1-22.

8. Kirkpatrick S, Gelatt CD, Jr., Vecchi MP. Optimization by simulated annealing. *Science*. 1983;220:671-680. doi: 10.1126/science.220.4598.671

9. Hastie T. Ridge Regularization: An Essential Concept in Data Science. *Technometrics*. 2020;62:426-433. doi: 10.1080/00401706.2020.1791959

10. Shokhirev MN, Johnson AA. Modeling the human aging transcriptome across tissues, health status, and sex. *Aging Cell*. 2021;20:e13280. doi: 10.1111/acel.13280

11. Johnson AA, Shokhirev MN, Lehallier B. The protein inputs of an ultra-predictive aging clock represent viable anti-aging drug targets. *Ageing Res Rev*. 2021;70:101404. doi: 10.1016/j.arr.2021.101404

12. Liang H, Zhang F, Niu X. Investigating systematic bias in brain age estimation with application to post-traumatic stress disorders. *Hum Brain Mapp*. 2019;40:3143-3152. doi: 10.1002/hbm.24588

13. Lu AT, Binder AM, Zhang J, Yan Q, Reiner AP, Cox SR, Corley J, Harris SE, Kuo PL, Moore AZ, et al. DNA methylation GrimAge version 2. *Aging (Albany NY)*. 2022;14:9484-9549. doi: 10.18632/aging.204434

14. Braun PR, Han S, Hing B, Nagahama Y, Gaul LN, Heinzman JT, Grossbach AJ, Close L, Dlouhy BJ, Howard MA, 3rd, et al. Genome-wide DNA methylation comparison between live human brain and peripheral tissues within individuals. *Transl Psychiatry*. 2019;9:47. doi: 10.1038/s41398-019-0376-y

15. Konigsberg IR, Barnes B, Campbell M, Davidson E, Zhen Y, Pallisard O, Boorgula MP, Cox C, Nandy D, Seal S, et al. Host methylation predicts SARS-CoV-2 infection and clinical outcome. *Commun Med (Lond)*. 2021;1:42. doi: 10.1038/s43856-021-00042-y

16. Boroni M, Zonari A, Reis de Oliveira C, Alkatib K, Ochoa Cruz EA, Brace LE, Lott de Carvalho J. Highly accurate skin-specific methylome analysis algorithm as a platform to screen and validate therapeutics for healthy aging. *Clin Epigenetics*. 2020;12:105. doi: 10.1186/s13148-020-00899-1

17. Dong Q, Song N, Qin N, Chen C, Li Z, Sun X, Easton J, Mulder H, Plyler E, Neale G, et al. Genome-wide association studies identify novel genetic loci for epigenetic age acceleration among survivors of childhood cancer. *Genome Med*. 2022;14:32. doi: 10.1186/s13073-022-01038-6

18. Choudhury A, Magill ST, Eaton CD, Prager BC, Chen WC, Cady MA, Seo K, Lucas CG, Casey-Clyde TJ, Vasudevan HN, et al. Meningioma DNA methylation groups identify biological drivers and therapeutic vulnerabilities. *Nat Genet*. 2022;54:649-659. doi: 10.1038/s41588-022-01061-8

19. Sturm G, Karan KR, Monzel AS, Santhanam B, Taivassalo T, Bris C, Ware SA, Cross M, Towheed A, Higgins-Chen A, et al. OxPhos defects cause hypermetabolism and reduce lifespan in cells and in patients with mitochondrial diseases. *Commun Biol*. 2023;6:22. doi: 10.1038/s42003-022-04303-x

20. Devall MA, Sun X, Eaton S, Cooper GS, Willis JE, Weisenberger DJ, Casey G, Li L. A Race-Specific, DNA Methylation Analysis of Aging in Normal Rectum: Implications for the Biology of Aging and Its Relationship to Rectal Cancer. *Cancers (Basel)*. 2022;15. doi: 10.3390/cancers15010045

21. Soliai MM, Kato A, Helling BA, Stanhope CT, Norton JE, Naughton KA, Klinger AI, Thompson EE, Clay SM, Kim S, et al. Multi-omics colocalization with genome-wide association studies reveals a context-specific genetic mechanism at a childhood onset asthma risk locus. *Genome Med*. 2021;13:157. doi: 10.1186/s13073-021-00967-y

22. Muse ME, Bergman DT, Salas LA, Tom LN, Tan JM, Laino A, Lambie D, Sturm RA, Schaider H, Soyer HP, et al. Genome-Scale DNA Methylation Analysis Identifies Repeat Element Alterations that Modulate the Genomic Stability of Melanocytic Nevi. *J Invest Dermatol*. 2022;142:1893-1902 e1897. doi: 10.1016/j.jid.2021.11.025

23. Thrush KL, Higgins-Chen AT, Liu Z, Levine ME. R methylCIPHER: A Methylation Clock Investigational Package for Hypothesis-Driven Evaluation &amp; Research. *bioRxiv*. 2022:2022.2007.2013.499978. doi: 10.1101/2022.07.13.499978

24. Levine ME, Lu AT, Quach A, Chen BH, Assimes TL, Bandinelli S, Hou L, Baccarelli AA, Stewart JD, Li Y, et al. An epigenetic biomarker of aging for lifespan and healthspan. *Aging (Albany NY)*. 2018;10:573-591. doi: 10.18632/aging.101414

25. Horvath S, Oshima J, Martin GM, Lu AT, Quach A, Cohen H, Felton S, Matsuyama M, Lowe D, Kabacik S, et al. Epigenetic clock for skin and blood cells applied to Hutchinson Gilford Progeria Syndrome and ex vivo studies. *Aging (Albany NY)*. 2018;10:1758-1775. doi: 10.18632/aging.101508

26. Zhang Q, Vallerga CL, Walker RM, Lin T, Henders AK, Montgomery GW, He J, Fan D, Fowdar J, Kennedy M, et al. Improved precision of epigenetic clock estimates across tissues and its implication for biological ageing. *Genome Med*. 2019;11:54. doi: 10.1186/s13073-019-0667-1

27. McEwen LM, O'Donnell KJ, McGill MG, Edgar RD, Jones MJ, MacIsaac JL, Lin DTS, Ramadori K, Morin A, Gladish N, et al. The PedBE clock accurately estimates DNA methylation age in pediatric buccal cells. *Proc Natl Acad Sci U S A*. 2020;117:23329-23335. doi: 10.1073/pnas.1820843116

28. Phipson B, Oshlack A. DiffVar: a new method for detecting differential variability with application to methylation in cancer and aging. *Genome Biol*. 2014;15:465. doi: 10.1186/s13059-014-0465-4

29. Shokhirev MN, Johnson AA. An integrative machine-learning meta-analysis of high-throughput omics data identifies age-specific hallmarks of Alzheimer's disease. *Ageing Res Rev*. 2022;81:101721. doi: 10.1016/j.arr.2022.101721

30. Liao Y, Wang J, Jaehnig EJ, Shi Z, Zhang B. WebGestalt 2019: gene set analysis toolkit with revamped UIs and APIs. *Nucleic Acids Res*. 2019;47:W199-W205. doi: 10.1093/nar/gkz401

31. Breitkreutz BJ, Stark C, Reguly T, Boucher L, Breitkreutz A, Livstone M, Oughtred R, Lackner DH, Bahler J, Wood V, et al. The BioGRID Interaction Database: 2008 update. *Nucleic Acids Res*. 2008;36:D637-640. doi: 10.1093/nar/gkm1001

32. Gene Ontology C, Aleksander SA, Balhoff J, Carbon S, Cherry JM, Drabkin HJ, Ebert D, Feuermann M, Gaudet P, Harris NL, et al. The Gene Ontology knowledgebase in 2023. *Genetics*. 2023;224. doi: 10.1093/genetics/iyad031
